# Antimicrobial applications of amphiphilic gold nanoparticles for antibiotic delivery

**DOI:** 10.1039/d5tb00961h

**Published:** 2025-09-16

**Authors:** Harita Yedavally, Matteo Gasbarri, Jan Maarten van Dijl, Francesco Stellacci, Anna Salvati

**Affiliations:** a University of Groningen, Department of Nanomedicine and Drug Targeting, Groningen Research Institute of Pharmacy Groningen The Netherlands a.salvati@rug.nl; b Institute of Materials, Ecole Polytechnique Fédérale de Lausanne Lausanne Switzerland; c University of Groningen, University Medical Center Groningen, Department of Medical Microbiology Groningen The Netherlands

## Abstract

Nanomedicine can offer novel strategies in the fight against antimicrobial resistance. Nano-sized drug carriers can be used to deliver antibiotics to their target to treat infections and some nanomaterials themselves have antimicrobial properties. Here, small amphiphilic gold nanoparticles with mixed ligand surfaces have been investigated for their potential use against bacterial infections in different settings. Owing to their unique surface properties, these nanoparticles are known to directly penetrate cell membranes, instead of entering cells by energy-dependent mechanisms of endocytosis, as observed for most nanomaterials. Therefore, we aimed to explore whether this capacity could be exploited to target and eliminate bacteria. To this end, different antibiotic-loaded small amphiphilic gold nanoparticles were prepared and their antimicrobial activity against the human pathogen *Staphylococcus aureus* was demonstrated. Next, we tested whether the antibiotic-loaded nanoparticles could be used to treat intracellular *S. aureus* infections, as well as to penetrate and eradicate biofilms. In the case of intracellular infections, nanoparticle uptake was accompanied by a mild decrease in the intracellular bacterial population. In the case of biofilms, instead, the nanoparticles were able to penetrate throughout the thickness of the biofilm, rather than only reaching the upper layers, as observed for most nanomaterials. Moreover, both the amphiphilic gold nanoparticles themselves and the antibiotic-loaded variants strongly induced death of biofilm-embedded bacteria.

## Abstract 2

Small amphiphilic gold nanoparticles with mixed ligand surfaces were loaded with antibiotics. The nanoparticles showed antimicrobial activity against *Staphylococcus aureus* and were able to penetrate biofilms and eradicate the bacteria.

## Graphical abstract

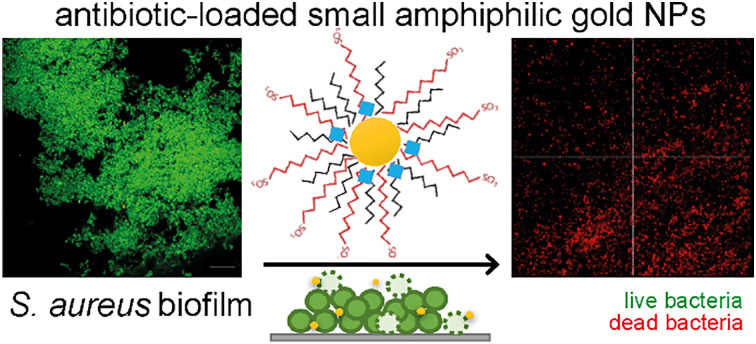

## Introduction

Antimicrobial resistance (AMR) is one of the biggest problems facing us today. Predictions suggest that the AMR-related deaths may outnumber those caused by cancer in the next 30 years.^1^ Pathogens are increasingly becoming resistant to the available drugs, forcing the use of higher doses or late-stage antibiotics like carbapenems. The faster evolution rate of microorganisms combined with their versatility and capability for rapid horizontal gene transfer compounds the problem. As long as we develop antibiotic molecules, there will be a subset of the bacterial population that will develop resistance to it. This has been evident throughout the history of the introduction of antibiotics into the market, where we have witnessed the emergence of resistant microorganisms within a few years, if not immediately.^2^ Therefore, there is a need for constant innovation to be able to continue treating infections caused by these pathogens.

Novel strategies are being explored constantly to deal with AMR, an overview of which is summarized in different reviews in the literature.^3^ Among the investigated alternatives, nanomedicine has been receiving increasing attention and may offer novel solutions for different applications. Indeed, the use of nanomaterials in the biomedical field has grown in recent decades, thanks to the improvement in technology for manipulating materials at the nanoscale.^4^ Thus, it is only natural that several studies investigated the potential use of nanomaterials against AMR.^5–8^ For instance, nano-sized materials can be used as carriers to deliver drugs to hard-to-reach places or to improve their delivery efficiency.^9–12^ This is particularly attractive for improving the efficacy of existing antibiotics with poor solubility and stability, as well as in the case of intracellular infections.^13,14^ Several bacteria have the capacity to enter cells and hide inside them, hijacking the existing cellular pathways.^15–17^ After entering the cells, the internalized bacteria in some cases can remain confined inside cellular organelles along the endo-lysosomal pathway, while in other cases, they can escape such organelles and reach the cytosol.^18–22^ Either way, treating intracellular bacteria is difficult because of the often-limited capacity of many antibiotics to pass the cell membrane and reach the intracellular location where the bacteria reside. In similar cases, by taking advantage of the unique capacity of nanoparticles (NPs) to enter cells, nano-sized drug carriers can be used to load and deliver antibiotics efficiently inside cells. This can be coupled to different targeting strategies to guide the NPs to specific organs and cells. A better delivery and higher efficacy are key in reducing the risk of the generation of resistance.

Next to similar applications for the delivery of antibiotics, nanomaterials can also be used to coat surfaces to prevent microbial fouling or biofilm formation. Biofilms form when planktonic bacteria adhere to a surface, then form a monolayer and start to produce a matrix that encapsulates them.^23–25^ This layer of polysaccharides, proteins and extracellular DNA provides protection to the biofilm-embedded bacteria, allowing them to persist physical and chemical insults. Penetration of molecules through this dense layer is difficult and thus, high concentrations of antibiotics are needed to eradicate biofilm-embedded bacteria. Additionally, treatments usually manage to reach only the bacteria in the upper biofilm layer, because of poor drug penetration, leaving the deeper-seated bacteria untouched, allowing the biofilm to propagate. Previous studies using NPs with amphiphilic mixed ligands^26^ showed that they can release their hydrophobic drug load at an acidic pH. This may actually provide opportunities to target biofilm-embedded bacteria with antibiotic-loaded NPs, because the pH inside a biofilm is relatively low. In fact, the environment inside biofilms can be quite different from that inside cells, as well as from that of planktonic bacteria.

Interestingly, some nanomaterials themselves can be used for their antimicrobial properties, such as silver NPs, which can release silver ions due to their partial solubility.^3,15^ Another material that has been studied extensively for different applications in nanomedicine, as well specifically against AMR, is gold. Gold nanoparticles (AuNPs) have found extensive applications in the biomedical field, including nanomedicine and AMR, because of their unique electronic and optical properties, as well as chemical inertness.^27–30^ Although their fate in the body after administration needs to be carefully considered, they are generally considered to be biocompatible.^27,30,31^ Other key advantages of AuNPs are their versatility and the ease of adding multiple surface functionalities.^16,32,33^ In fact, in-solution synthesis methods to control the size, shape, and surface functionality of AuNPs were developed as early as 1951,^34^ and they have subsequently been modified several times for tailored applications. Among these examples, some of the authors^35^ studied small amphiphilic AuNPs (roughly 3 nm diameter) coated with mixed surface ligands and showed that, in contrast to what is observed for most nanomaterials, which are internalized by cells *via* energy-dependent uptake mechanisms,^36,37^ these NPs are able to enter cells in an energy-independent manner.^38^ This unique capacity has already been exploited to deliver drugs intracellularly in applications related to cancer^26^ and immunotherapy.^39^ Recently, it has been demonstrated that AuNPs with mixed ligands also show broad-spectrum antiviral properties^40^ and are effective against bacteriophages.^41^ Another advantage of these particles is that small molecular drugs can be easily loaded on the surface of the NPs within the surface ligands, without chemical modification.

Owing to these unique properties, in this study, we sought to investigate the use of amphiphilic AuNPs with mixed ligands against bacteria, bacterial infections and biofilm-embedded bacteria. *Staphylococcus aureus* is notorious for being one of the more problematic pathogens and features on the ESKAPE list issued by the World Health Organization (WHO), which includes the major multidrug-resistant pathogens that plague us today.^15^*S. aureus* is a highly versatile pathogen that can exist in a variety of configurations, including intracellular locations and biofilms.^21^ Thus, in this study, we aimed to investigate whether the small size and unique surface properties of amphiphilic AuNPs, and their ability to traverse cell membranes in an energy-independent manner, could be exploited in different AMR settings. First, we studied whether these NPs could directly enter the bacteria because of their capacity to penetrate lipid bilayers. Next, we tested their use for the delivery of antibiotics to treat intracellular infections, using an endothelial cell barrier model and a lung epithelial cell barrier model, that were previously established.^18,19^ Finally, we tested the capacity of the AuNPs – bare and antibiotic-loaded – to penetrate and treat biofilms.

## Materials and methods

### Nanoparticle synthesis

AuNPs were synthesized according to previously established protocols.^42^ Briefly, gold salt and the two surface ligands, 11-mercaptoundecane sulfonate (MUS) and 1-octanethiol (OT), or MUS and 3,5-dimethyl-1-octanethiol (br-OT), were dissolved in ethanol at a feed ratio of 2 : 1. Then, 200 ml of 50 mM NaBH_4_ in ethanol was added dropwise to the mixture over 2 h, where the NPs precipitated out. The NPs were washed several times with ethanol using centrifugation and then with water using Amicon ultra 1530 K centrifugal devices. The particles were then freeze-dried and stored as a powder until further use.

To achieve fluorescence labeling of the MUSOT NP, a BODIPY 630/650-X NHS ester (Invitrogen) was thiolated through the succinimidyl ester group to yield BODIPY-SH. A stock solution of this in acetone was added at a 50-fold molar excess to MUSOT in water and stirred in the dark over 48 h at 25 °C to allow the exchange reaction. The particles were then washed 5 times with acetone as previously described to remove unreacted dye and, lastly, the particles were freeze dried.^42^

### Nanoparticle characterization

Transmission electron microscopy was used to estimate the NP size distribution. A drop of the NP at a concentration of 0.1 μg ml^−1^ was deposited on a 400-mesh copper grid coated with a carbon support and allowed to dry. Images were acquired with an FEI Talos electron microscope equipped with a Ceta CCD camera at an acceleration voltage of 200 kV. At least 100 particles were analyzed from each batch of synthesized NPs, and their size was calculated using a custom macro in ImageJ.

Hydrogen NMR spectroscopy was used to assess the purity of the NP and to characterize the ratio of the different ligands on the surface of the NP. As the assembly of the surface ligands on the NP causes broadening of their NMR peaks, the absence of sharp peaks in the NMR spectrum confirmed the absence of unbound ligands. To determine the ratio of the two ligands, the gold core of the NPs was etched using 20 mg ml^−1^ iodine in methanol-d4 and sonicated for 30 min before performing NMR spectroscopy. The ligand ratio was calculated using the integrals shown in SI Fig. S1.

### Drug loading

Six antibiotics were chosen to load onto the NPs – dicloxacillin, oxacillin, nafcillin, fusidic acid, isoniazid, and bronidox – by hydrophobic partitioning. The antibiotics were dissolved in organic solvents (ethanol, methanol, or acetone) and mixed with an aqueous solution of the NPs. The mixture was left overnight with constant stirring to allow the organic solvent to evaporate, causing the drug to settle in hydrophobic pockets on the NP surface. Unbound drug was washed with water using Amicon 30k centrifugal filters. To estimate the amount of loaded drug, the gold core of the NPs was etched using a 10 mg ml^−1^ KCN solution in methanol, followed by analysis using a UV-vis spectrophotometer against antibiotic standard curves. The amount of unbound drug in the washes was also monitored with a UV-vis spectrophotometer and the sample was washed until no unbound drug was detected. The NP conjugates were stored as a concentrated 10 mg ml^−1^ aqueous solution.

### Bacterial culture

An overnight culture of *Staphylococcus epidermidis* ATCC 35984 or *Staphylococcus aureus* HG001 was diluted to an optical density at 600 nm (OD_600_) of 0.1 in tryptic soy broth (TSB) and seeded in a 96-well flat-bottomed plate along with a concentration series of the synthesized amphiphilic AuNPs, antibiotics, and NP–antibiotic conjugates. The bacterial growth was monitored over 18 h by measuring the OD_600_ using a plate reader (Biotek) at 37 °C with constant shaking at 250 rpm. Growth curves were plotted and the inhibitory concentration IC_50_ was determined for each treatment.

### Transmission electron microscopy

An overnight culture of *S. aureus* HG001 was diluted to an OD_600_ of 0.1 in TSB and incubated with 10 μg ml^−1^ of MUS:OT AuNPs for 1 h at 37 °C with constant shaking at 250 rpm. The bacteria were fixed using 4% paraformaldehyde/2% glutaraldehyde in 0.1 M sodium cacodylate buffer (pH 7.4) for 30 min and pelleted into 4% low melting agarose. The bacteria-containing agarose pellet was cut into approximately 2 mm^3^ pieces to allow further processing. The pellet was post-fixed in 1% osmium tetroxide/1.5% potassium ferrocyanide in 0.1 M sodium cacodylate for 1 h. The pellet was then washed with MQ water, a graded ethanol series was used for dehydration, and finally the pellet was embedded in EPON resin, which was allowed to polymerize at 58 °C for 24–48 h. Thin sections were cut using a standard diamond knife on a UC7 ultramicrotome (Leica, Vienna, Austria). Images were recorded using a Biotwin CM100 transmission electron microscope (FEI, Eindhoven, The Netherlands) operated at 80 kV using a Morada digital camera. Energy dispersive X-ray scattering was acquired using an X-max 150 detector (Oxford Instruments) on a Supra 55 scanning electron microscope (Zeiss) to confirm the presence of the gold core of the MUS:OT NP.

### Cell culture and infection experiments

Infection experiments with primary human umbilical vein endothelial cells (HUVECs) from pooled donors (Lonza) were performed as previously described.^18,43^ Briefly, the cells were seeded at 3000 cells cm^−2^ in a multi-well plate and grown to form a barrier over 7 days in Endothelial Growth Medium 2 (EGM2, PromoCell), with the medium changed every alternate day. Infection experiments with 16HBE14o- lung epithelial cells, a transformed bronchial epithelial cell line originally derived from a 1-year-old heart-lung transplant patient,^44^ were performed as previously described.^19,45^ Briefly, the cells were seeded at 10^5^ cells cm^−2^ in a multi-well plate and cultured for 48 h in minimum essential medium (MEM) supplemented with 1% (v/v) nonessential amino acids 100× and 2% (v/v) l-glutamine 200 mM (Gibco, the Netherlands). All cells were incubated at 37 °C in a humidified incubator with 5% CO_2_.

To establish an intracellular infection, a GFP-expressing strain of *S. aureus* HG001 was used. To constitutively express GFP, the bacteria carried plasmid pJL-sar-GFP.^46^ Bacteria were harvested during the mid-exponential growth phase (OD_600_ of 0.4).

For both cell lines, the invasion assay was performed by allowing bacteria at a multiplicity of infection (MOI) of 25 to invade the cells, after which the extracellular bacteria were eliminated using 25 μg ml^−1^ of lysostaphin (AMBI Products LLC, Lawrence, NY) for 1 h. Different antibiotics, amphiphilic NPs, and NP–antibiotic conjugates were added to the cells at 8 h post-infection (p.i.) in the case of 16HBE14o-, and the cells were collected for further analysis by flow cytometry at 24 h and 48 h p.i. In the case of the HUVEC, the treatments were performed at 24 h post-infection, and cells were collected at 48 h and 72 h p.i. for further analysis.

### Flow cytometry

To prepare samples for flow cytometry analysis, after exposure to the bacteria and/or NPs and antibiotics, cells were washed twice with phosphate-buffered saline (PBS), detached with trypsin, spun down at 300 g, fixed in 4% formalin for 30 min, and resuspended in 100 μl of PBS for analysis. The cell suspension was analyzed with a CytoFLEX S flow cytometer (Beckman Coulter, The Netherlands). The gating strategy was as follows: an SSC-A *vs.* FSC-A double scatter plot was used to select the cell population and exclude debris, followed by an SSC-H *vs.* SSC-A double scatter plot to exclude doublets. The GFP-expressing bacteria were detected in the FITC channel (excitation laser 488 nm, emission filter 525/40 nm) in a FITC-A *vs.* SSC-A plot. The fraction of cells that contained bacteria was monitored, as well as the intensity of their GFP signal. Data analysis was performed using Kaluza 2.0 software (Beckman Coulter).

### Biofilm targeting

For biofilm formation, an overnight culture of *S. epidermidis* ATCC 35984 or *S. aureus* HG001 was diluted to an OD_600_ of 0.1 in TSB. A 300 μl aliquot of the diluted bacteria was then seeded in an 8-well glass chamber slide and incubated at 37 °C to allow biofilm formation. After 24 h, the floating planktonic bacteria on the surface were removed and the medium was replaced with TSB containing varying concentrations of antibiotics, amphiphilic AuNPs, or NP–antibiotic conjugates. The concentrations employed were 25, 250, and 500 μg ml^−1^ for the NP–antibiotic conjugates, 100 μg ml^−1^ for the antibiotics, and 500 μg ml^−1^ for the amphiphilic AuNPs. After a further 24 h of incubation at 37 °C, the biofilms were washed twice with PBS to remove planktonic bacteria and excess NPs and stained using a live/dead BacLight Bacterial Viability Kit (Thermo Fisher). Briefly, this kit contains two dyes, SYTO™ 9 (Ex/Em 485/498 nm), which penetrates both viable and nonviable bacteria, and propidium iodide (Ex/Em 535/617 nm), which only penetrates bacteria with damaged membranes, quenching the fluorescence of SYTO 9 in the process. Images were taken in multiple *z* planes with a Leica SP8 confocal laser scanning microscope (Leica, Wetzlar, Germany) to gain a complete picture of the biofilm structure. Images were analyzed using LAS X software (Leica) and ImageJ software.

### Data analysis

Data analysis was performed using GraphPad Prism version 8 (GraphPad Software, CA, USA).

## Results and discussion

### Nanoparticle synthesis and antibiotic loading

**Fig. 1:**
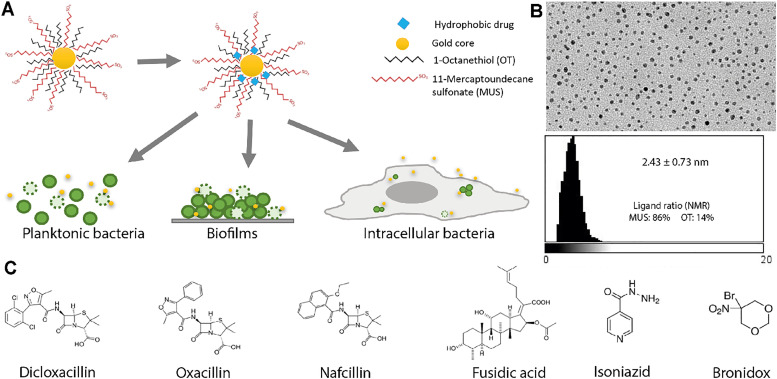
Drug loading on gold nanoparticles (AuNPs; adapted from the study by Mottas *et al.*).^26^ (A) Hydrophobic antibiotics were loaded onto synthesized amphiphilic AuNPs and tested on different systems: planktonic bacteria, biofilms, and intracellular bacteria. (B) Characterization of the AuNP size using transmission electron microscopy. The ratios of the two surface ligands, 11-mercaptoundecane sulfonate (MUS) and 1-octeanethiol (OT), as measured by ^1^H-NMR spectroscopy, were 86% and 14%, respectively. (C) Chemical structure of the antibiotics loaded onto the MUSOT AuNP: dicloxacillin, oxacillin, nafcillin, fusidic acid, isoniazid, and bronidox.

AuNPs with the surface ligands, MUS and OT (Fig. 1A), were synthesized and characterized according to previously described methods.^12^ Some experiments were performed with particles containing br-OT, and these are discussed later in the text. TEM was used to determine the NP size. For this, several images were collected at a magnification of at least 64 000× and a custom ImageJ macro was run to measure the size distribution of the particles, as shown in Fig. 1B. The results showed that the gold cores had an average size of 2.43 ± 0.73 nm, with a very narrow distribution. ^1^H-NMR spectroscopy was performed to check the purity of the synthesized NPs, to ensure that there were no peaks from unbound ligands present (SI Fig. S1A). Binding of the ligands on the NP surface causes broadening of the NMR peak, while unbound ligands show up as sharp peaks. Once the purity of the NP was confirmed, the ratio of the two ligands on their surface was determined by etching the gold core of the NP using iodine, acquiring the ^1^H-NMR spectra, and using the NMR spectrum to calculate the relative amounts (SI Fig. S1B). A more detailed overview of the applied calculations is presented in the study by Guven *et al.*^42^ The obtained MUS:OT NMR ratio of 86 : 14 in response to a stoichiometric feed ratio of 66 : 34 is in line with previous studies on synthesizing these NPs.^41^

Previous studies showed that the AuNPs can be loaded with small hydrophobic drug molecules, and these drug-loaded NPs were quite successful in the delivery of anti-cancer drugs.^26^ Thus, here, we hypothesized that these NPs could also be used as carriers for antibiotics against bacteria. A big advantage of these particles is that the drug loading is physical, so there is no need for chemical modification of the drugs. The drugs that can be loaded onto the particles are limited based on the size, owing to the small size of the particles, and hydrophobicity, due to the composition of the surface ligands. To this end, we selected 6 antibiotics of small size and high hydrophobicity and loaded them onto the gold NPs by simple hydrophobic partitioning. For this, an aqueous solution of the NPs was mixed with the antibiotic dissolved in an organic solvent (acetone, methanol or ethanol depending on the antibiotic), which was allowed to evaporate overnight under constant stirring. This led the drug to settle in the hydrophobic pockets in between the surface ligands as the solvent evaporated. Several washing steps were performed to ensure that any excess unbound drug was removed. This was also confirmed using UV-vis spectroscopy on the washes. The amount of drug loaded onto the particles was measured against calibration curves using UV-vis spectroscopy after etching the gold core using cyanide. Some optimization was necessary to determine the suitable organic solvent used, the ratio of the particles and the drug used, as well as the number of washes to avoid loss of material. This varied for the different drugs, with some being more effectively loaded onto the particles. Overall, all selected antibiotics could be successfully loaded, with a loading amount in the range of ∼10% by weight for dicloxacillin and bronidox to ∼20% for isoniazid and fusidic acid (Table 1). It would be interesting to study more in detail what makes the loading of certain molecules more effective, and further optimization could also be performed to potentially improve the loading efficiency of the antibiotics.

**Table 1:** Drug loading and IC_50_ concentrations of the NP conjugates

Antibiotic	Drug loading, wt%	Free drug IC_50_, μg ml^−1^	NP–drug IC_50_, μg ml^−1^
Dicloxacillin	10.0	0.185	2.992
Oxacillin	13.0	4.510	0.463
Nafcillin	12.2	0.497	1.072
Fusidic acid	20.4	0.175	0.504
Isoniazid	20.0	21.850	∼0.0975
Bronidox	10.3	∼1.444	3.325

Having confirmed drug loading, we then tested the effect of the conjugates against the same amount of free drugs on free bacteria, intracellular bacteria, and bacterial biofilms, all of which are discussed in what follows.

### Interaction of NPs with bacteria and antimicrobial effects of antibiotic-loaded NPs

**Fig. 2:**
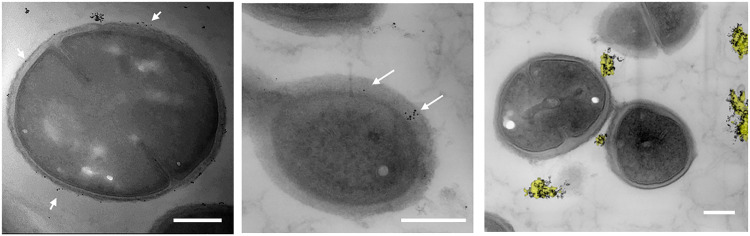
TEM images of *S. aureus* with MUSOT NPs. Arrows indicate NPs interacting with the peptidoglycan layer of the bacterium. In the right panel, energy-dispersive X-ray spectroscopy (EDX) overlay mapping with gold shown by the yellow overlay, confirming the NPs’ gold core. Scale bar: 200 nm.

As a first step, we sought to see the effect of the NPs and the NP–drug conjugates on planktonic bacteria. Initial studies showed that the amphiphilic MUS:OT NPs did not have a toxic effect on the Gram-positive bacterium tested, namely, *S. aureus* (SI Fig. S2). Only at excessive concentrations (>1000 μg ml^−1^) did the particles retard bacterial growth. To visualize the interaction, *S. aureus* was exposed to 100 μg ml^−1^ of MUS:OT NPs and transmission electron microscopy was performed, as shown in Fig. 2. Energy dispersive X-ray analysis confirmed that the particles we observed were in fact gold (Fig. 2, right panel). We noticed that these particles interacted mainly with the bacterial surface and accumulated on it. In some cases, some particles seemed to partially penetrate the outer peptidoglycan layer (an example is shown in the central image of Fig. 2), but no particles were observed inside the bacteria. Overall, these results suggest that the unique capacity of the AuNPs to penetrate the cell membrane of eukaryotic cells^7^ does not extend to the investigated bacteria. A better understanding of the precise mechanisms by which these NPs can enter lipid membranes may help to unravel the different behavior observed in this case.

**Fig. 3:**
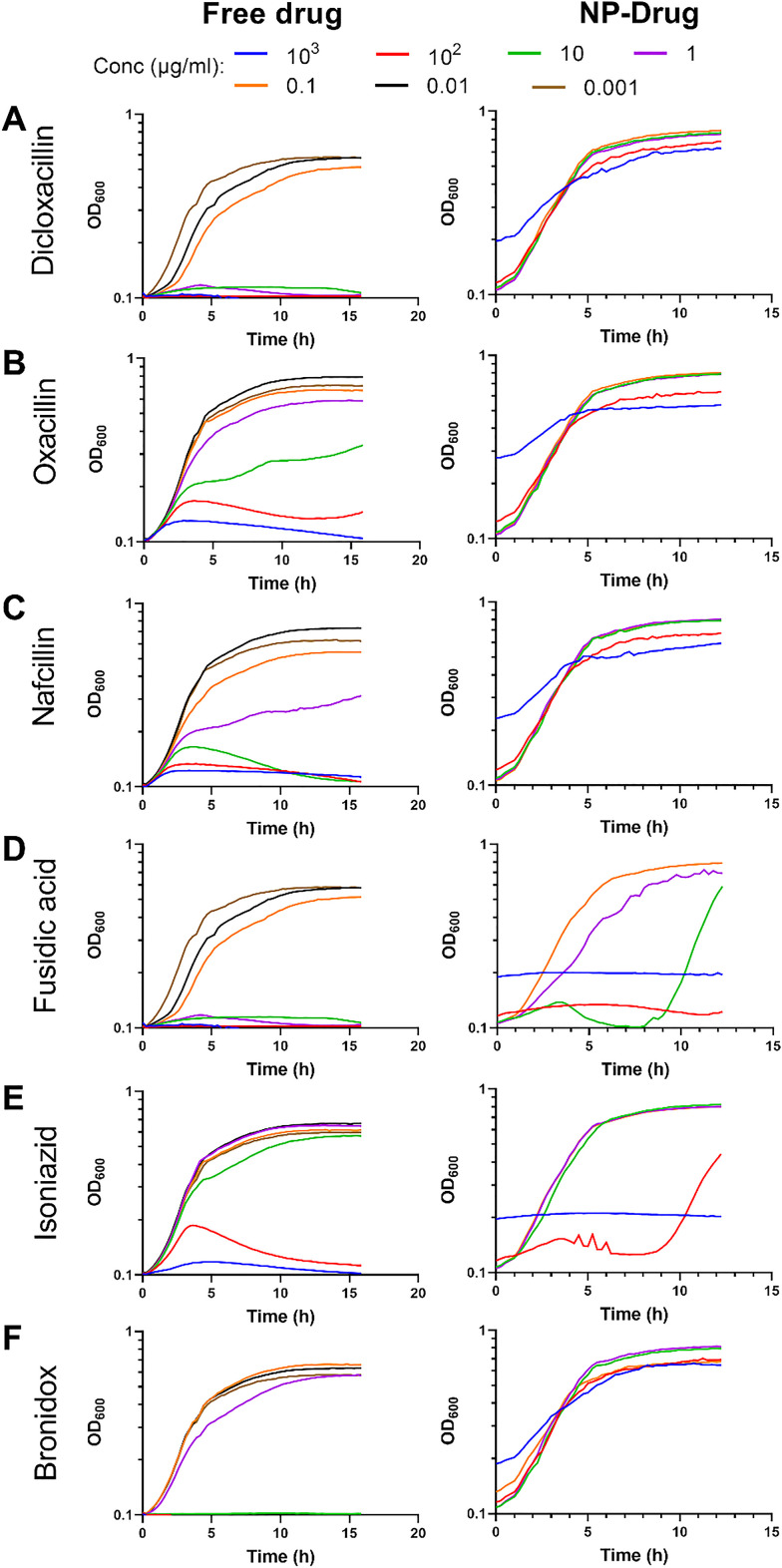
Effect of NP–antibiotic conjugates on the growth of planktonic bacteria. Growth curves of *S. epidermidis* treated with different concentrations of 6 antibiotics and the corresponding NP–drug conjugates for dicloxacillin (A), oxacillin (B), nafcillin (C), fusidic acid (D), isoniazid (E) and bronidox (F).

Next, we sought to test the NP conjugates loaded with different antibiotics to see if they had any effect on bacteria as compared to the free drug. To this end, we treated *S. epidermidis* with a concentration series of the NP conjugates and corresponding free antibiotics (Fig. 3). Given that the loading efficiency onto the NPs was different for the different antibiotics, both the concentration of the NP conjugate and the corresponding amount of drug present in it are shown in Table 1. The results showed that all tested conjugates were able to either kill the bacteria or retard their growth to varying degrees in a concentration-dependent manner. Based on these results, the concentrations of the conjugates and free antibiotics needed to inhibit bacterial growth by 50% (IC_50_) were calculated and are shown in Table 1. From this, we conclude that varying levels of success were achieved, with dicloxacillin, nafcillin, fusidic acid and bronidox conjugated to AuNPs being less effective than the free drugs. On the other hand, oxacillin and especially isoniazid were much more effective when conjugated to the AuNPs than as free drugs.

To understand the above observations, it is important to keep in mind that the drugs are loaded onto the particles by physical interaction forces only, and it is unclear what conditions cause them to be released from the core. Previous studies showed that this could occur on interaction with the lipid bilayer or in response to changes in the environment. For instance, Mottas *et al.* noticed that the drug was released from the NPs at a lower pH.^26^ Bacterial growth overnight causes a change in the pH of the medium around it. It could be interesting in future studies to determine whether the NPs have different effects on planktonic bacteria in more pH regulated environments. Thus, the exact release mechanism for each NP conjugate would need to be investigated individually in order to explain the differences observed for the different antibiotics.

In summary, the drugs were loaded onto the particles, albeit with different loading efficiencies. The MUS:OT NPs by themselves did not have an effect on the planktonic growth of bacteria and loading the drugs onto the particles had differential effects on the efficacy of the drugs. These differences and, in particular, the lower efficacy which was observed in some cases in comparison to the free drug are likely connected to the different capacity of each drug to detach from the NPs to exert its action.

To achieve optimal loading and improve delivery, the drug should remain on the drug carrier – here the NP's ligand shell – until it reaches its target. Yet, in order for the drug to be effective, a subsequent release from the carrier is required. Further tuning on the NP design and choice of antibiotics is needed to achieve the ideal balance between these partially contrasting requirements. For instance, the loading efficiency could potentially be improved by using different ratios of the surface ligands, given that the hydrophobicity of the NPs changes based on this. The ratio of surface ligands could also affect the (potential) release of the cargo. Likewise, other antibiotics could be tested for similar applications. Importantly, even in cases where the effect on planktonic bacteria was in the same order of magnitude for the free and NP conjugated antibiotics, drug efficacy should also be compared in more complex settings, where many other parameters affect the capacity of the drug or NP conjugate to reach the bacteria, as well as the capacity of the drug to release from the NPs to exert its action. Therefore, as a next step, we tested the NP conjugates on different models of intracellular infections.

### Interaction of NPs with cells: uptake and antimicrobial effects of antibiotic-loaded NPs in intracellular infection models

As the amphiphilic AuNPs used here were successfully applied in previous studies to deliver small molecule drugs into cells for applications against cancer,^26,39^ we hypothesized that they could be used also against intracellular bacteria. Intracellular bacteria are often difficult to address with antibiotics and can cause recurrent infections in several diseases.^47^ Thus, we tested the particles on two established intracellular infection models, namely, 16HBE14o- lung epithelial cells that have been used to model pulmonary respiratory infection conditions,^19^ and primary umbilical vein endothelial cells (HUVECs) that have been used to model diseases like endocarditis.^48,49^ Both these cell lines have been used to establish well-characterized intracellular infection models with the same strain of *S. aureus*, as used for this study.^18,19^

Using fluorescently labelled MUS:OT NPs (with bodipy-630/650) and sodium azide to deplete the cell energy, we first confirmed by flow cytometry that these NPs can enter both HUVECs and 16HBE14o- cells in an energy-independent manner (SI Fig. S3).^38^ In agreement with this, electron microscopy imaging confirmed the presence of the amphiphilic NPs both inside and outside membrane-bound compartments, as expected due to their capacity of entering cells passively (SI Fig. S4).^38^

**Fig. 4:**
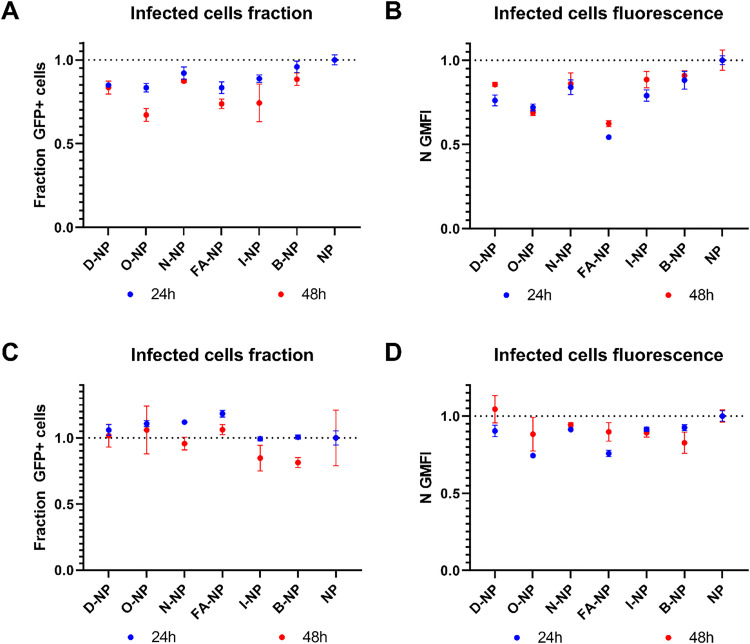
Effect of NP conjugates on intracellular GFP-expressing *S. aureus* in (A), (B) HUVECs and (C) and (D) 16HBE140- cells, as measured by flow cytometry at 24 h and 48 h post-infection. Cells were treated with the 6 different NP–drug conjugates at a concentration of 50 μg ml^−1^. (A) and (C) show the effects of drug-loaded NPs on the fraction of infected cells relative to cells treated with the plain NPs, and (B) and (D) show the GFP intensity, representing the bacterial population density inside the infected cells. NP–D, dicloxacillin–NP; NP–O, oxacillin–NP; NP–N, nafcillin–NP; NP–FA, fusidic acid–NP; NP–I, isoniazid–NP; NP–B, bronidox–NP; and NP, plain NP.

Next, HUVECs were grown to a monolayer barrier over 7 days^18,43^ and infected with GFP-expressing *S. aureus* (see the Methods section for details). The infection progress in HUVECs is shown in SI Fig. S5. In this model, the intracellular bacteria are present inside membrane-enclosed compartments through the course of the infection. Similarly, 16HBE14o- cells were grown to form a barrier as previously described^19^ and infected with GFP-expressing *S. aureus*. In this model, the intracellular *S. aureus* bacteria reach the cytoplasm starting from 6 h post-infection (SI Fig. S6). At 24 h or 8 h post-infection, various treatments of NPs, free antibiotic, or NP conjugates were added to the HUVEC and 16HBE14o- barriers. In particular, the 6 NP conjugates were tested at a NP concentration of 50 μg ml^−1^ on the two barrier models and compared to the corresponding amounts of free antibiotic. At 24 h and 48 h post-infection of both the HUVEC and 16HBE14o- barriers, the fraction of cells containing GFP-expressing bacteria (which shows the number of infected cells) and the intensity of the GFP signal in those cells (which reflects the number of bacteria inside them) were measured by flow cytometry (Fig. 4). Of note, dead bacteria may still show GFP fluorescence and hence, the GFP intensity alone may actually underestimate the antibacterial activity of the gold conjugates. For HUVECs, we noticed that all of the tested conjugates had an effect on lowering both the fraction of infected cells and the intracellular bacterial population, as reflected by the cell fluorescence. However, the effects on the number of infected cells were relatively small in the conditions tested. Further studies will be required to determine whether potential quenching from the internalized gold could contribute in part to the observed reduction in GFP intensity and overall to improve intracellular delivery. For the 16HBE14o- cells, instead, only minor effects on the intracellular bacterial population were observed. As these cells have a large volume, with relatively few internalized bacteria per cell, it is conceivable that at the concentrations tested, the particles simply did not reach the bacteria in high enough amounts or possibly the antibiotic release from the NPs that did reach the bacteria was still too low.

We anticipate that further optimization of the conjugates in relation to antibiotic loading and release from the core, as well as optimization of the surface ligand ratio or the NP core size, may allow us to achieve higher efficacy against these intracellular bacteria. Similarly, conjugating the particles to a targeting receptor, as previously shown,^39^ could also further increase their uptake in the infected cells and, hence, the amount of intracellular antibiotic.

### Interaction of NPs with biofilms: biofilm penetration and antimicrobial effects of bare and antibiotic-loaded NPs

As a next step, we investigated the effects of NP conjugates in a different configuration, namely, bacteria in biofilms. Importantly, the pH inside biofilms is known to be relatively low. This may be advantageous since previous studies using NPs with amphiphilic mixed ligands^26^ showed that they can release their hydrophobic drug load at an acidic pH. In addition, the small size of the NPs and the capacity to penetrate lipid bilayers might enable them to also penetrate through biofilms. Thus, we hypothesized that the NP conjugates could potentially have an effect on biofilms, and we therefore sought to test this. Next to the MUS:OT gold NPs, we also tested NPs with a similar composition, with the exception that the OT ligand on the surface was branched at the C3 and C7 positions, instead of being linear (here referred to as branched NPs or br-NPs). These branched NPs enter cells through the conventional endo-lysosomal pathway^38^*via* an active uptake mechanism.

Biofilms of *S. epidermidis* were grown in 8-chamber slides over two days, with the treatment being added 24 h after the bacterial seeding. This allowed sufficient time to form a distinct biofilm on the glass surface. Non-adherent bacteria were washed away, and the biofilm was stained with a live–dead viability kit and, subsequently, inspected using confocal microscopy. The live–dead kit uses two dyes, SYTO-9 that enters and stains both live and dead bacteria, and propidium iodide that can enter only dead bacteria, thereby quenching the fluorescence of SYTO-9.

**Fig. 5:**
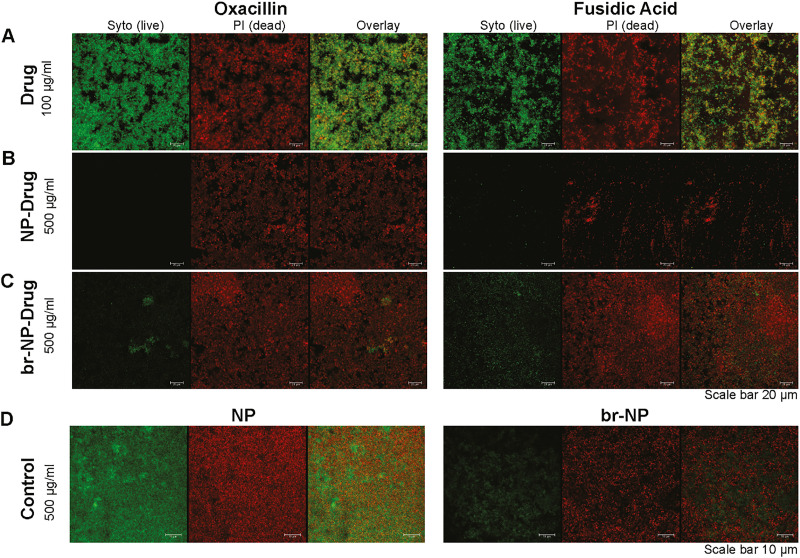
Effect of drug conjugates on *S. epidermidis* biofilms. Biofilms were formed in 8-chamber glass chamber slides over 48 h. 24 h after seeding, different treatments were added to the biofilms: (A) oxacillin and fusidic acid at 100 μg ml^−1^ (drug), (B) NP–drug conjugates at 500 μg ml^−1^, (C) br-NP–drug conjugates at 500 μg ml^−1^, and (D) bare NPs and br-NPs at 500 μg ml^−1^ (control). Biofilms were visualized after a further 24 h using the live/dead viability kit. Live bacteria were stained by SYTO 9, shown in green, and dead bacteria by propidium iodide, shown in red.

*S. epidermidis* is known for its high ability to form biofilms and over the course of 48 h, such biofilms can reach up to 10 μm in thickness. Since penetration to the lower layers of biofilms is usually a challenge, we sought to see how deep the NPs could penetrate into the biofilms, which we tested by exposing them to bodipy-630/650 conjugated MUS:OT NPs. A *z*-projection of a 3D *z*-stack image by confocal microscopy showed that, as we hypothesized, indeed the particles penetrated all the way to the bottom of the biofilm (SI Fig. S7). Next, we tested the two conjugates that showed an effect on internalized bacteria in HUVECs, fusidic acid and oxacillin, with the two types on nanoparticles, NP and br-NP, starting with a fairly high concentration of 500 μg ml^−1^. The effects were compared to those observed with the corresponding amount of free antibiotics (100 μg ml^−1^) as well as the bare NPs (Fig. 5).

The results showed that the free antibiotics were partially effective against the biofilm at the concentration tested, as shown in Fig. 5A, where holes in the biofilm are visible, but many live bacteria are still present. Interestingly, when exposed to the unloaded NP and br-NP controls at 500 μg ml^−1^, some effects were also observed, stronger in the case of the br-NPs, where more holes in the biofilms were visible (Fig. 5). Substantial effects on the biofilms were observed with all four conjugates tested. In particular, both oxacillin conjugates (NP–O and br-NP–O at 500 μg ml^−1^) were very effective at killing the biofilm-embedded bacteria (Fig. 5B and C). In the case of the fusidic acid conjugates, NP–FA and br-NP–FA (500 μg ml^−1^), stronger effects were observed with br-NP–FA, with NP–FA being partially effective and br-NP–FA completely destroying the biofilm (Fig. 5B and C). Bare NPs tested at comparable concentrations only achieved a partial effect on the biofilms (Fig. 5D). We then tested lower concentrations (250 μg ml^−1^ and 25 μg ml^−1^, as shown in SI Fig. S8) to determine whether these effects would still be visible. The results showed a concentration-dependent effect for NP–O, with br-NP–O being most effective. NP–FA instead was effective at all concentrations tested, while only partial effects were observed for br-NP–FA at the lower concentrations.

**Fig. 6:**
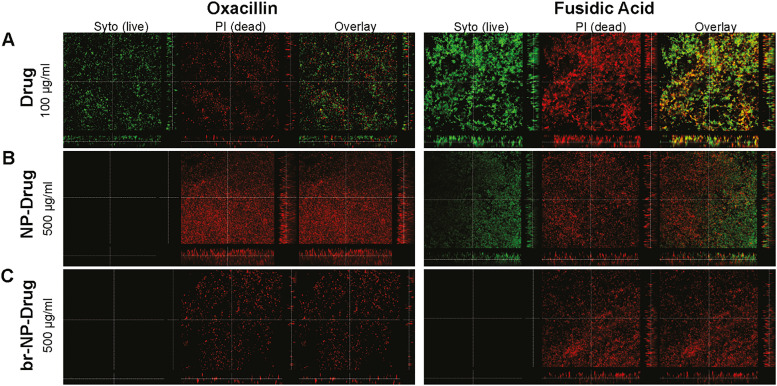
Effect of drug conjugates on *S. aureus* biofilms. Biofilms were formed in 8-chamber glass slides over 48 h. 24 h after seeding, different treatments were applied to the biofilms: free drug at 100 μg ml^−1^ (drug) or NP–drug and br-NP–drug conjugates at 500 μg ml^−1^. Biofilms were visualized after a further 24 h using the live/dead viability kit. Live bacteria are stained by SYTO 9, shown in green, and dead bacteria by propidium iodide, shown in red.

Comparable experiments were performed with *S. aureus*, the bacteria used in the intracellular models, to determine whether the different antibiotic–NP conjugates and particles are effective also on biofilms from another pathogen (Fig. 6). Also in this case, we again observed a greater effect of the NP–drug conjugates as compared to the free drugs. Both drug-loaded NPs were highly effective at destroying the biofilms and performed better than the free drug.

Overall, the results clearly showed that the antibiotic-loaded AuNPs were very effective at combating biofilms. Even when partially effective, the thickness of the biofilm was greatly reduced upon treatment with the conjugates and several holes were visible. We therefore conclude that the NPs could be used in combination with other drugs to fully eliminate the remaining bacteria, taking advantage of their capacity to penetrate the lower layers of the biofilm.

## Conclusions

The aim of the present study was to test small amphiphilic gold NPs for their applications against staphylococcal infections in different settings. The synthesis, which was conducted in line with earlier research, was successfully completed, and a series of antibiotics could be easily loaded onto the NPs using a straightforward process, taking advantage of the unique properties of their surface ligands (with varying loading efficiency for the different drugs).

Despite their capacity to penetrate lipid bilayers and eukaryotic cell membranes, when exposing *S. aureus* cultures to the amphiphilic NPs, no gold was observed inside the bacterial cells. This is perhaps not surprising given the different nature and composition of the bacterial cell envelope. When testing the different NP conjugates, instead, the effects on the bacterial growth curves were smaller or in some cases equal to what was observed with the same amount of free drug. This is likely connected to differences in drug release from the gold core. The NP design could be changed, for instance, by changing the ligand ratio, in order to optimize this aspect for each drug type and improve the respective drug release profiles.

We then tested the capacity of the mixed ligand NPs to reach intracellular bacteria applying two different infection models involving *S. aureus* internalization in endothelial or epithelial cells.^18,19^ Only in the endothelial cell model, some effects were observed in a number of internalized bacteria. However, the intracellular infections could not be eradicated in the conditions tested. Further optimization of the NP design may enable us to increase their uptake and drug release profile and hence their efficacy in targeting intracellular bacteria.

Instead, when the NPs were used to challenge biofilms, very strong biofilm-disruptive effects were observed, where the drug-loaded NPs penetrated the biofilms better than the free drug, thereby more effectively killing the biofilm-embedded bacteria. Two drugs were tested on biofilms formed with two types of bacteria. In all these cases, the conjugate NPs were effectively bactericidal, and even low concentrations of some of the NP–drug conjugates resulted in full eradication of bacteria. Preliminary studies (data not shown) suggest that these observations can extend to other Gram-positive bacteria, including methicillin resistant *S. aureus* (MRSA) strains as well. For future research, it would be interesting to test whether similar effects could also be obtained with biofilms formed by Gram-negative bacteria, such as *Acinetobacter baumannii* or *Pseudomonas aeruginosa*.

Overall, the strong effects observed on biofilms treated with NP conjugates are likely connected to their observed capacity to penetrate the full thickness of the biofilm and possibly to efficient drug release within the biofilm microenvironment. Given these promising observations, further studies will be useful to further optimize the applications of amphiphilic AuNP drug conjugates in different infection settings.

## Conflicts of interest

The authors have no conflicts of interest to declare.

## Supplementary Material

TB-013-D5TB00961H-s001

## Data Availability

The datasets included in the current study are available from the corresponding author upon reasonable request. Supplementary information is available, including additional nanoparticle characterization, effect of nanoparticles on *S. aureus*, uptake of nanoparticles by cells in standard and energy-depleted conditions, and additional electron and fluorescence microscopy images of infected human cells and biofilms incubated with the nanoparticles. See DOI: https://doi.org/10.1039/d5tb00961h.
