# Supplementary material for: Antimicrobial applications of amphiphilic gold nanoparticles for antibiotic delivery
Source: J Mater Chem B. 2025 Sep 16;13(42):13769–80. doi: 10.1039/d5tb00961h (PMC12495389; doi:10.1039/d5tb00961h)
Supplement: TB-013-D5TB00961H-s001 [file TB-013-D5TB00961H-s001.pdf]

Supplementary Information (SI) for Journal of Materials Chemistry B.
This journal is © The Royal Society of Chemistry 2025

Supplementary information

Antimicrobial applications of amphiphilic gold nanoparticles for antibiotic delivery

Harita Yedavally, Matteo Gasbarri, Jan Maarten van Dijl, Francesco Stellacci, Anna Salvati*

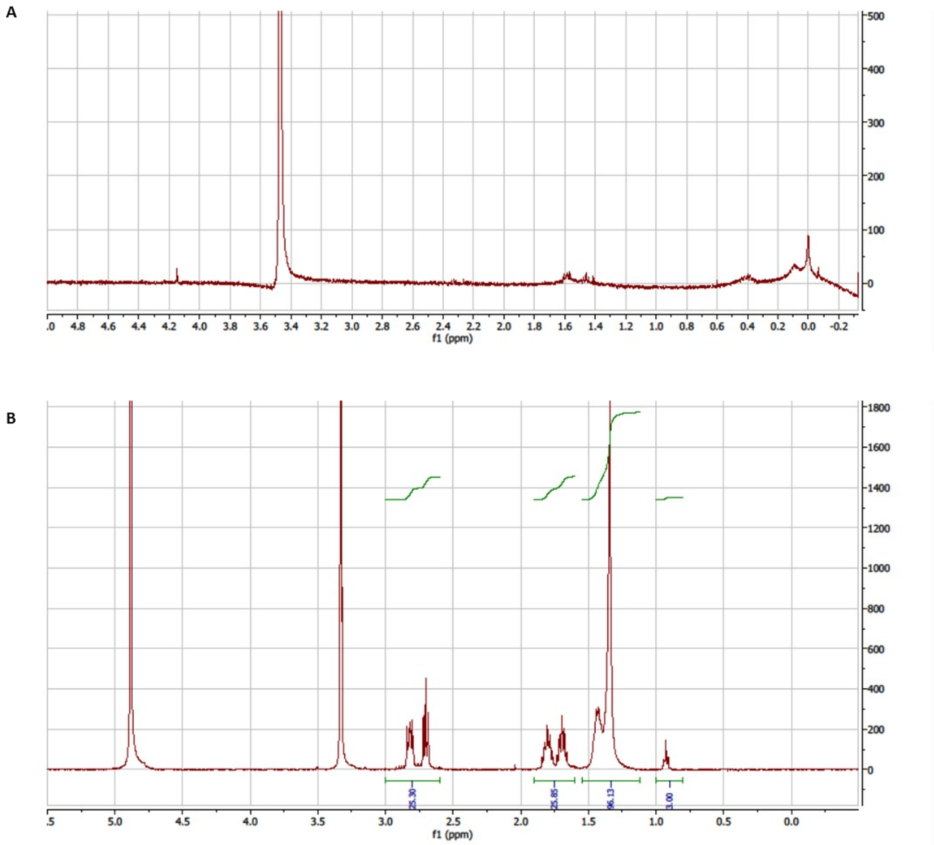

	Range	Normalized	Absolute
1	3.00 .. 2.60	25.30	89531.62
2	1.90 .. 1.60	25.85	91475.07
3	1.55 .. 1.12	96.13	340149.58
4	1.00 .. 0.80	3.00	10609.84

Supplementary Figure S1. MUSOT NP characterization **(A)** ^{1}H-NMR spectrum to analyze the purity of the synthesized MUSOT NPs, where the peak broadening and absence of any sharp peaks demonstrate that there are no free unbound ligands present. **(B)** ^{1}H-NMR spectrum of the ligands released after etching the gold core of the synthesized NPs. The ratio of the two ligands is assessed from the peaks, and measured to be 86% and 14% of MUS and OT, respectively.

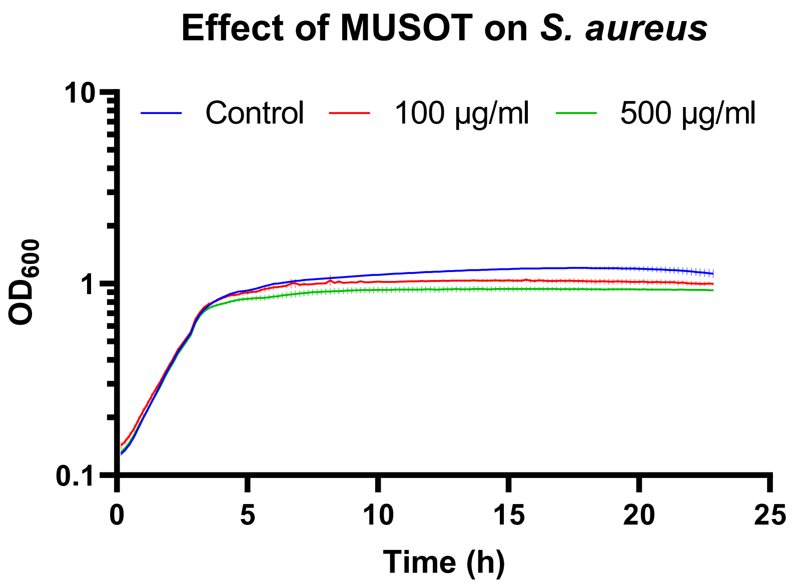

Supplementary Figure S2. *S. aureus* HG001 was treated with 100 or 500 µg/ml MUSOT NPs, or no NPs (control), and the growth was monitored over 24 h by OD_{600} readings. The particles did not detectably affect the bacterial growth, even at high concentrations.

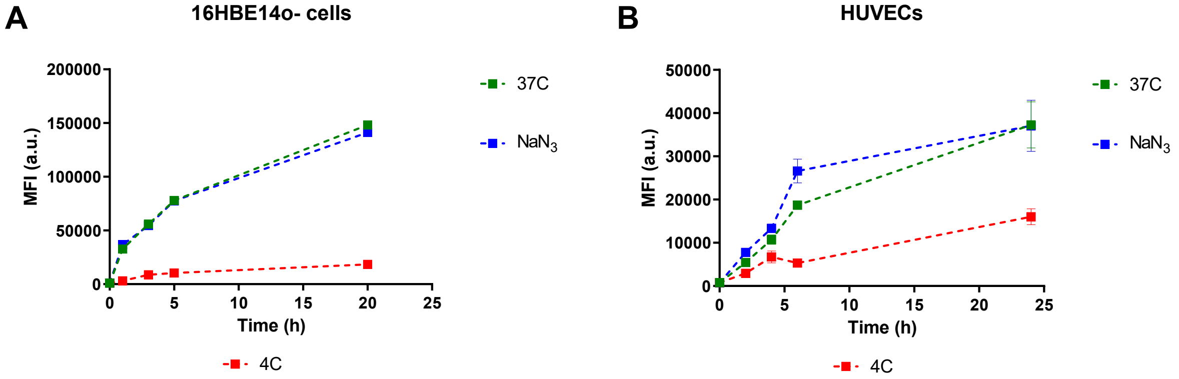

Supplementary Figure S3. Uptake of Bodipy-630/650 MUS:OT NPs by **(A)** HUVEC and **(B)** 16HBE14o- cells measured by flow cytometry. MUSOT NPs at a concentration of 50 µg/ml were added to cells in 3 conditions: in standard conditions at 37°C, or 4°C where uptake is blocked, and at 37°C with sodium azide, where the cell's energy is depleted and active uptake through endocytosis is blocked. The uptake of NPs at 37°C by cells incubated with or without sodium azide was comparable, showing that the NPs entered the cells passively, as previously shown.(1) Interestingly, instead, at 4°C the NP uptake was blocked, likely due to a lower membrane bilayer fluidity at low temperature, which hinders the NP penetration.

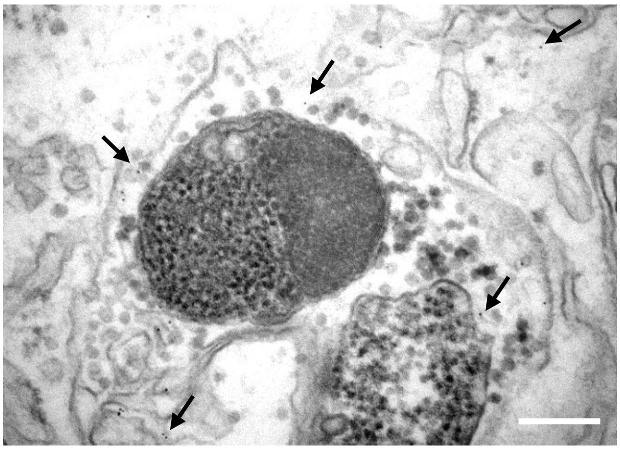

Supplementary Figure S4. Transmission electron microscopy of intracellular bacteria in HUVEC 24 h post infection. All bacteria (darker electron dense areas) were observed in membrane-enclosed compartments. MUSOT NPs are visible both inside and outside these membrane-enclosed compartments, demonstrating that the NPs have access to the entirety of the cell, as expected due to their known capacity to enter cells passively.(1) Scale bar: 100 nm.

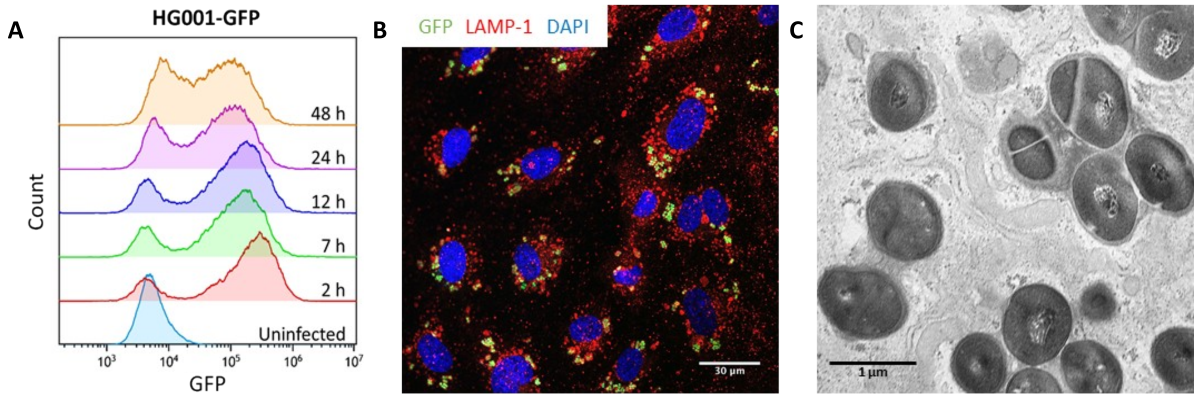

Supplementary Figure S5. Intracellular infection model based on HUVEC. **(A)** Cell fluorescence distributions measured by flow cytometry of HUVEC infected with *S. aureus* HG001-GFP, presenting the progression of infection at different times post addition of the bacteria. The plots show the fraction of host cells without bacteria (not-infected, left peak) and infected cells (right peak) based on the intensity of GFP, as measured by flow cytometry. Under the applied conditions, bacteria are found in roughly 80% of the cells and persist inside the cells up to 48 h post infection. **(B)** Confocal microscopy of a HUVEC barrier infected by *S. aureus* HG001-GFP at 7 h post infection, showing GFP-expressing bacteria (green) inside LAMP-1 immuno-stained lysosomes (red). Blue: DAPI-stained nuclei. Scale bar: 30 µm. **(C)** Transmission electron microscopy image showing intracellular electron-dense (black) *S. aureus* inside HUVEC cells at 7 h post infection in multiple membrane-enclosed compartments, as clusters or individual bacteria. Scale bar: 1 µm. In this model, all bacteria are found in membrane-enclosed subcellular compartments, which most likely represent compartments along the endo-lysosomal pathway. No bacteria are observed free in the cytosol.(2)

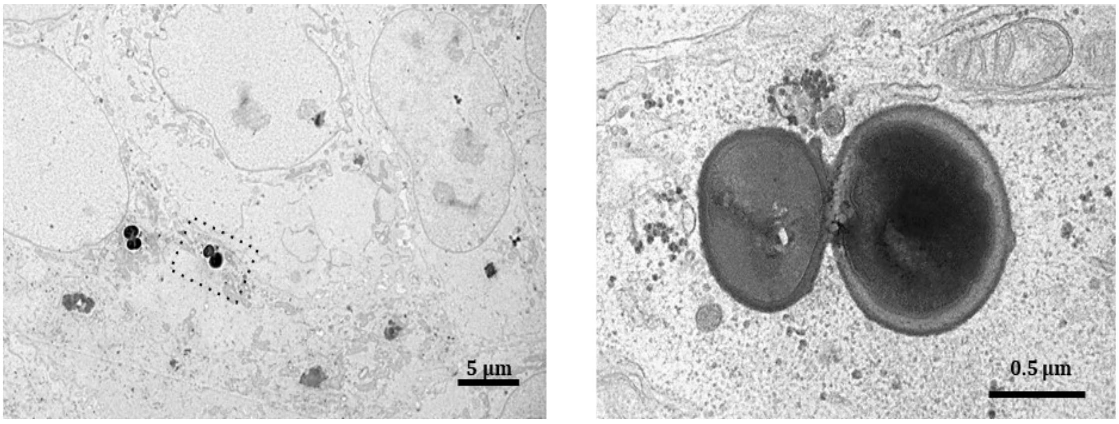

Supplementary Figure S6. Transmission electron microscopy of intracellular bacteria in 16HBE14o- cells 24 h post infection. All bacteria (darker electron dense areas) were found free in the cytoplasm (no membrane enclosing them is visible).(3)

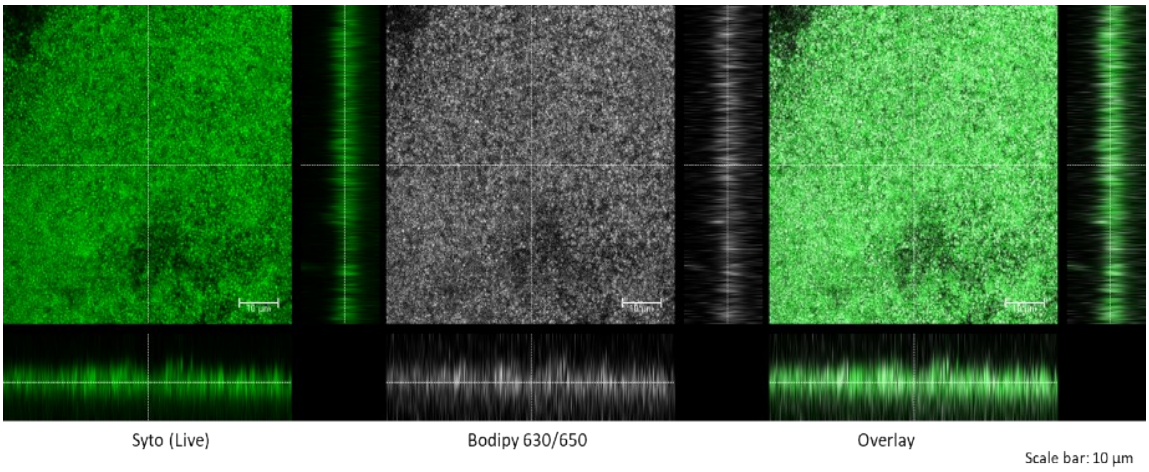

Supplementary Figure S7. Penetration of MUSOT NPs in an *S. epidermidis* biofilm. Bodipy-630/650 labelled MUSOT NPs were added to a *S. epidermidis* biofilm 24 h after seeding, and imaged a further 24 h later. Live bacteria were stained by Syto 9, shown in green, and the NPs are shown in white. As evident from the z-projection, the NPs penetrated the biofilm all the way to the bottom.

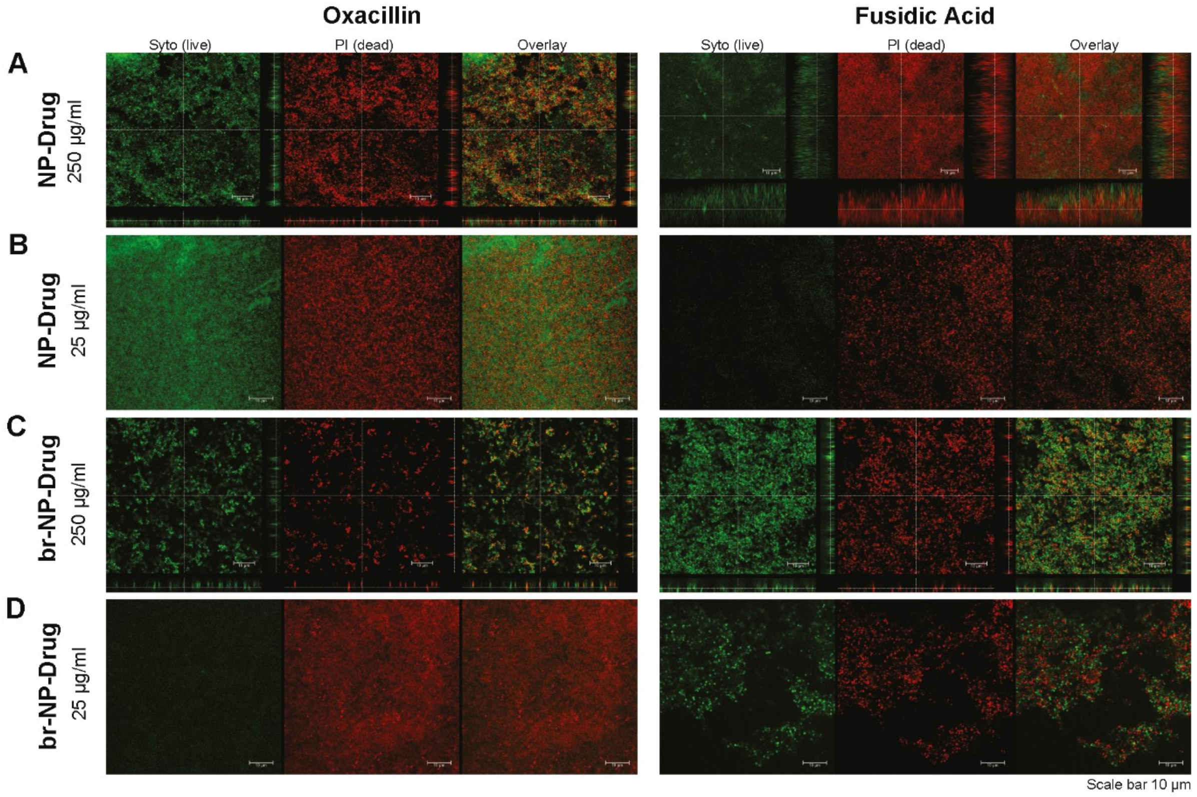

Supplementary Figure S8. Effects of NP-drug conjugates on *S. epidermidis* biofilms at different concentrations. Biofilms were formed in 8-chamber glass slides over 48 h. 24 h after seeding, different treatments were added to the biofilms, including NP-Drug conjugates **(A-B)** and br-NP-Drug conjugates **(C-D)** at 250 or 25 µg/ml. Biofilms were visualized after a further 24 h incubation using the Live/Dead viability kit. Live bacteria were stained by Syto 9, shown in green, and dead bacteria by propidium iodide, shown in red. Scale bar: 10 µm.

References

1. Verma A, Uzun O, Hu Y, Hu Y, Han HS, Watson N, et al. Surface-structure-regulated cell-membrane penetration by monolayer-protected nanoparticles. Nat Mater. 2008;7(7):588-95.

2. Raineri EJM, Yedavally H, Salvati A, van Dijl JM. Time-resolved analysis of Staphylococcus aureus invading the endothelial barrier. Virulence. 2020;11(1):1623-39.

3. Palma Medina LM, Becker AK, Michalik S, Yedavally H, Raineri EJM, Hildebrandt P, et al. Metabolic Cross-talk Between Human Bronchial Epithelial Cells and Internalized Staphylococcus aureus as a Driver for Infection. Mol Cell Proteomics. 2019;18(5):892-908.
